# Lipid-Based Natural Food Extracts for Effective Control of Botrytis Bunch Rot and Powdery Mildew on Field-Grown Winegrapes in New Zealand

**DOI:** 10.3390/plants10030423

**Published:** 2021-02-24

**Authors:** Kirstin V. Wurms, Annette Ah Chee, Peter N. Wood, Joseph T. Taylor, Frank Parry, Robert H. Agnew, Duncan Hedderley, Philip A. G. Elmer

**Affiliations:** 1 The New Zealand Institute for Plant and Food Research Limited (Plant & Food Research), Private Bag 3230, Waikato Mail Centre, Hamilton 3240, New Zealand; Annette.AhChee@plantandfood.co.nz (A.A.C.); Joseph.Taylor@plantandfood.co.nz (J.T.T.); Frank.Parry@plantandfood.co.nz (F.P.); Philip.Elmer@plantandfood.co.nz (P.A.G.E.); 2 Plant & Food Research, Private Bag 1401, Havelock North 4157, New Zealand; Peter.Wood@plantandfood.co.nz; 3 Plant & Food Research, P.O. Box 845, Blenheim 7240, New Zealand; Rob.Agnew@plantandfood.co.nz; 4 Plant & Food Research, Private Bag 11600, Palmerston North 4442, New Zealand; duncan.hedderley@plantandfood.co.nz

**Keywords:** anhydrous milk fat, *Botrytis cinerea*, *Erysiphe necator*, integrated pest management, natural products, soybean oil, vineyard

## Abstract

Synthetic controls of crop pathogens are increasingly associated with harm to the environment and human health, and pathogen resistance. Pesticide residues in crops can also act as non-tariff trade barriers. There is therefore a strong imperative to develop biologically based and natural product (NP) biofungicides as more sustainable alternatives for crop pathogen control. We demonstrate the field efficacy, over multiple seasons, of NP biofungicides, NP1 (based on anhydrous milk fat) and NP2 (based on soybean oil), on two major diseases of winegrapes—Botrytis bunch rot (Botrytis) and powdery mildew (PM). The NPs were integrated into a season-long integrated disease management programme that has produced chemical-residue-free wines. Efficacies for Botrytis control on three different varieties were: 63–97% on Chardonnay, 0–96% for Sauvignon Blanc and 46–58% on Riesling; with 65–98% PM control on Chardonnay and Riesling. NP2 exhibited the significant control of Botrytis latent infections, making it a viable alternative to mid-season synthetic fungicides. Disease control was significantly better than the untreated control and usually as efficacious as the synthetic fungicide treatment(s). Yields and wine quality in NP-treated crops were normally equivalent to those in the synthetic fungicide treatments. The results indicate that NP-mediated disease control of Botrytis and powdery mildew can be obtained in the vineyard, without synthetic fungicide input.

## 1. Introduction

Wines are among the most high-value and widely consumed horticultural products in the world [1]. In New Zealand, they are the second most lucrative horticultural export behind kiwifruit [2]. Globally, the most devastating and ubiquitous diseases of grapes are Botrytis bunch rot (Botrytis) and Powdery Mildew (PM) [3,4], caused by the fungal pathogens *Botrytis cinerea* and *Eryspihe necator* (also known as *Uncinula necator*), respectively.

*Botrytis cinerea*, a pathogen that is both necrotrophic and saprophytic, favours cooler, moist climates and causes both yield losses and wine taints. It can be difficult to control because it utilises a range of different infection pathways [5]. For example, latent endophytic infection and infection from colonised dead floral tissue within the grape bunch, once it closes, make control difficult because the fungus is within an ideal microenvironment which is less permeable to topical fungicides [5]. Moreover, it infects a wide variety of different plant families and is very adaptable at developing resistance to conventional fungicides, such as dicarboximides, benzimidazoles, succinate dehydrogenase inhibitors, anilinopyrimidines, and quinone outside inhibitors (QoI, formerly known as strobilurins) [6].

Powdery mildew is a polycyclic disease caused by different biotrophic pathogens from the Erysiphales order, with each particular pathogen being host specific. It flourishes in warmer, dryer climes [7]—although the sexual stage requires free moisture to release ascospores from its highly robust chasmothecia in spring, secondary spread through asexual conidia requires only high atmospheric humidity. This pathogen can infect all aerial parts of the grape plant, causing yield loss and reducing wine quality [8]. It can be treated with sulphur, but this natural product can be phytotoxic to some grape varieties [9] and has been associated with respiratory and eye irritation with chronic exposure in humans [10]. PM is already resistant to several members of the synthetic fungicide groups such as benomyl, demethylation inhibitors (DMIs), and QoI [11]. Furthermore, the teleomorphic stage, a key source of inoculum for the next season, is becoming much more prevalent in commercial New Zealand vineyards [12] and is directly associated with increased disease severity [13]. 

In addition to the problems associated with resistance to synthetic fungicides, the application of products throughout the growing season has now become restricted because of global demands for residue-free wine, and many wineries have adopted "residue-free" fungicide spray programmes. This strategy has required residue-causing fungicides to be applied much earlier in the growing season (e.g., pre-bunch closure), often leaving the fruit inadequately protected during the mid and late parts of the season, when berries become increasingly susceptible to Botrytis infection [5].

Natural products (NP) for bioprotection have been used around the world as effective alternatives to traditional synthetic pesticides because they offer the advantages of being more environmentally benign and more acceptable for human health [14,15,16,17,18]. The New Zealand Institute for Plant and Food Research Limited (PFR) have developed two biofungicides for the control of PM and Botrytis. NP1 is an emulsified concentrate of anhydrous milk fat (AMF), obtained from dairy cows; NP2 is an emulsified concentrate of pure soybean oil. The formulations are based on naturally derived fats/oils, and only contain co-formulants that are derived from the human food industry and are, therefore, considered to have low toxicity or have Generally Regarded As Safe (GRAS) status. Disease control offered by mineral oils, along with oils derived from plants and animals, is also very durable [19,20]. Research to date has shown that NP1 and NP2 were highly effective against various PM pathogens on different crops in laboratory and glasshouse experiments [21,22,23,24,25]. However, these environments were artificially controlled, and product efficacy in a highly variable field environment was unknown, hence the need for the current research. Winegrapes were chosen as the exemplar crop because of their high value, the importance of Botrytis and PM to worldwide production, and because we specifically wanted a model crop that was grown in the open field rather than being produced under glass. The aims of this study were to demonstrate that effective disease control of these pathogens on winegrapes could be obtained in the field over a number of seasons, under different disease pressures, in different environments and on different grape varieties, and without adverse effects on yield.

This paper highlights that disease control of two key pathogens in vineyards is possible using fungicides based on natural products.

## 2. Results

**Figure 1:**
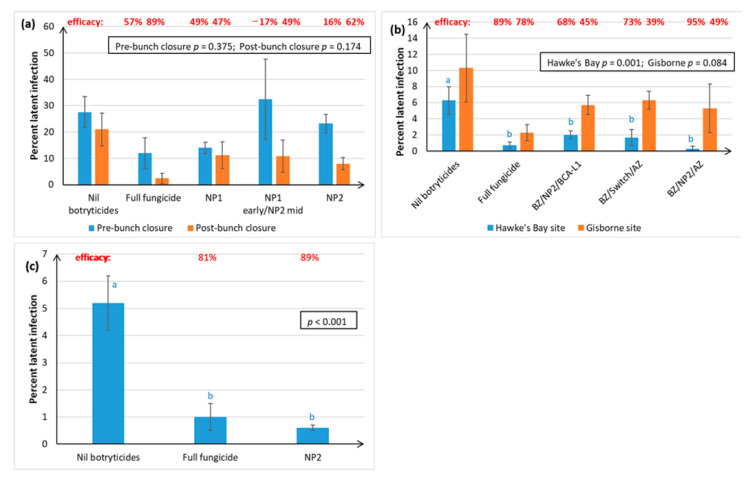
Percent incidence of Botrytis latent infections (latents) on immature berries from Chardonnay and Sauvignon Blanc grape varieties, following freezing and incubation in high humidity chambers. The efficacy (E) of each spray programme for Botrytis control (shown in red text) was calculated using the formula, E = ((U − T)/U) × 100, where T is the percentage of latents in the test treatment and U is the percentage disease in the Nil botryticide control. A negative E value indicates that disease is higher in the treatment than in Nil botryticide control. Treatments are described in full in Tables S1 and S2, but briefly comprised: Nil botryticides (negative control), where 1–3 sprays of a powdery mildew-specific fungicide were applied and no botryticides; full fungicide (positive control), where a mixture of fungicide products were applied at 7- to 21-day intervals from late-November through to mid-March to provide season long disease control; NP1 and NP2, which were applied 1–4× during the early- and mid-season of phenological development, i.e., between post-bloom and post-bunch closure. For the NP treatments, BOTRY-Zen® (BZ, 4 kg/ha formulation of the biocontrol agent, *Ulocladium oudemansii*) was also applied during the remainder of the early-season, and BCA-L1, an experimental formulation of *Aureobasidium pullulans* (2 × 10^7^ spores/mL) was applied during the late season (mid-February to mid-March) to provide season-long disease control using only biopesticide products. Switch® fungicide (800 g/ha) and ARMOUR-Zen (AZ, 5 L/ha formulation of chitosan) were sometimes used as alternative mid- and late-season treatment comparisons. Each lettered graph represents a separate experiment, set up in a randomised block design. Different ascending numbering systems are used for Chardonnay vs. Sauvignon Blanc experimental seasons to indicate that trials for both varieties were performed over consecutive seasons, but that season 1 in Chardonnay does not necessarily correspond to the same calendar year as Season i in Sauvignon Blanc. On each graph, error bars indicate standard errors, and the boxed values provide the probabilities obtained from analysis of variance (ANOVA). Different letters indicate statistically significant differences from pairwise likelihood ratio tests but are only presented when the ANOVA *p* ≤ 0.05. The concentration of the active ingredient in the NP formulation is indicated in parentheses for each specific experiment. (**a**) Chardonnay, Season 2, Hawke’s Bay site, with assessments at two times—pre-bunch closure and post-bunch closure, NP1 (7 g/L) and NP (15 g/L); (**b**) Chardonnay, Season 4, with two assessment sites—Hawke’s Bay and Gisborne, NP2 (5 g/L); (**c**) Sauvignon Blanc, Season i, Hawke’s Bay site, NP2 (5 g/L).

Earlier trials used both NP1 and NP2, but later trials focussed mainly on NP2 because the emulsion was more stable and gave more effective control of Botrytis latent infections at post-bunch closure (Figure 1a).

### 2.1. Botrytis Assessments

#### 2.1.1. Latent Infections

Latent infections (latents) were only assessed in two Chardonnay seasons (Figure 1a,b), and one of the Sauvignon Blanc seasons (Figure 1c). No assessments were made for Riesling.

Overall, efficacy of latent infection control for all treatments was better at post-bunch closure rather than pre-bunch closure (Figure 1a), so all subsequent assessments were made post-bunch closure. Apart from the NP1 early/NP2 mid treatment, all NP treatments reduced the incidence of latents relative to the control, although these reductions were only statistically significant when the percentage of latent infections in the Nil botryticide control was <10% (Figure 1b,c). Under these conditions, the NP2 treatment rivalled the full fungicide treatment.

#### 2.1.2. Infections at Vintage

**Figure 2:**
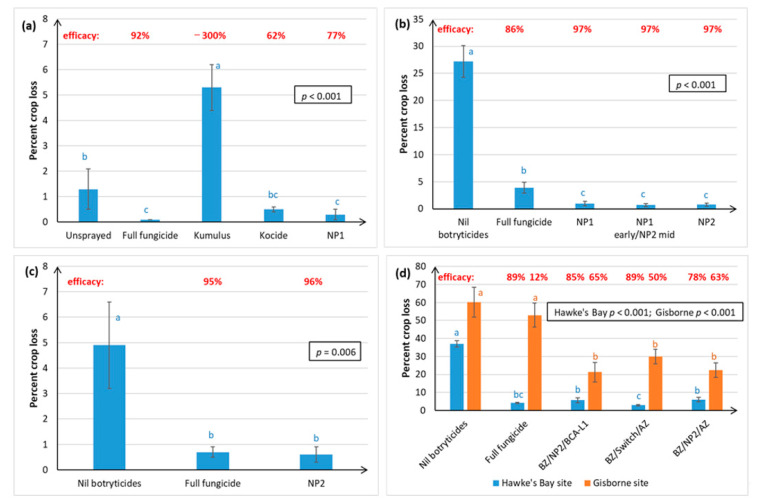
Percentage crop loss resulting from Botrytis bunch rot infections of Chardonnay grapes, as assessed at harvest in April, over four successive seasons. Crop loss was calculated as (%bunch severity × %bunch incidence)/100 for 50 randomly harvested bunches per plot. The efficacy calculation is outlined in Figure 1. Treatments are described in full in Tables S1 and S2, and briefly in Figure 1. In Season 1 only (Figure 2a only), there was an unsprayed treatment, and the Kumulus^®^ DF (3 g/L), Kocide^®^ 2000 DF and NP1 (7 g/L) treatments were applied right throughout the growing season (from mid-November until mid-March) instead of just between post-bloom and post-bunch closure (Figure 2**b**–**d**). Each graph represents data from a separate season, with error bars indicating standard errors, boxed values showing probabilities obtained from ANOVA, and different letters indicating statistically significant differences from pairwise likelihood ratio tests, which are only presented when the ANOVA *p* ≤ 0.05. The concentration of the active ingredient in the NP formulation is indicated in parentheses for each specific experiment. (**a**) Season 1, Hawke’s Bay site, NP1 (7 g/L); (**b**) Season 2, Hawke’s Bay site, NP1 (7 g/L) and NP (15 g/L); (**c**) Season 3, Hawke’s Bay site, NP2 (5 g/L); (**d**) Season 4, Hawke’s Bay and Gisborne sites, NP2 (5 g/L).

Figure 2 shows that, across 4 seasons, the NP treatments significantly reduced Chardonnay crop loss resulting from Botrytis bunch rots, and that disease control rivalled the full fungicide spray programme. The same results were obtained under low disease pressure, indicated by low crop loss in the Nil botryticide control (Figure 2a,c) and higher disease pressure (Figure 2b,d). Kumulus^®^ use appeared to predispose plants to *B. cinerea* (Figure 2a). NP1 and NP2 were equally effective in reducing crop loss at vintage (Figure 2b). The BZ/Switch/AZ and BZ/NP2/AZ treatments allowed for a direct comparison between Switch and NP2 use during the mid-season, with Switch use resulting in significantly lower crop loss than NP2 in Hawke’s Bay, but the two treatments were equally as effective in Gisborne (Figure 2d).

With the exception of the NP1 early/NP2 mid treatment, Botrytis disease control efficacy in Chardonnay with NP application ranged from 63–97% efficacy (Figure 2).

**Figure 3:**
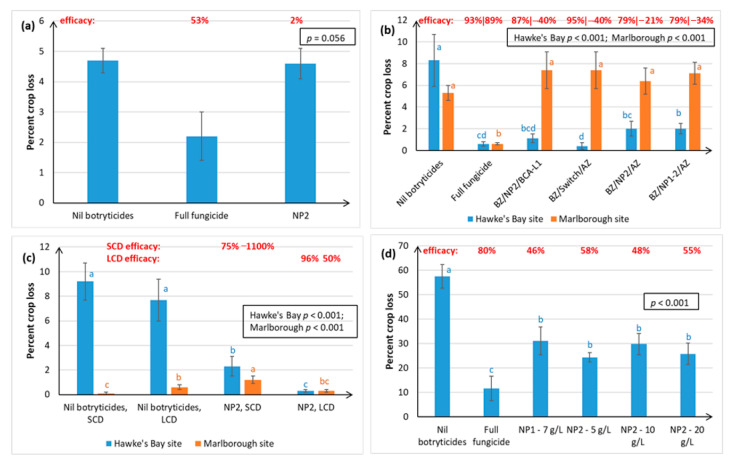
Percentage crop loss resulting from Botrytis bunch rot infections of Sauvignon Blanc (SB) and Riesling grape varieties, as assessed at harvest in April. Data were collected over 3 successive seasons for SB and one season for Riesling. Crop loss was calculated as described in Figure 2, and the efficacy calculation is outlined in Figure 1. Treatments are described in full in Tables S1 and S2, and briefly in Figure 1, except for the addition of treatments applied under different canopy densities, where leaf plucking and shoot thinning were used to create standard canopy densities (SCD), with bunch exposures of ca. 26%, and low canopy densities (LCD), with bunch exposures of ca. 80%. Each graph represents data from a separate trial, set up in a randomised block resign, with error bars indicating standard errors, boxed values showing probabilities obtained from ANOVA, and different letters indicating statistically significant differences from pairwise likelihood ratio tests, which are only presented when the ANOVA *p* ≤ 0.05. The concentration of the active ingredient in the NP formulation is indicated in parentheses for each specific experiment. (**a**) SB, Season i, Hawke’s Bay site, NP2 (5 g/L); (**b**) SB, Season ii, Hawke’s Bay and Marlborough sites, NP1 (5 g/L) and NP2 (5 g/L); (**c**) Season iii, Hawke’s Bay and Marlborough sites, new formulation of NP2 (30 mL/L); (**d**) Riesling, Hawke’s Bay, NP1 (7 g/L) and NP2 (5, 10 and 20 g/L).

Figure 3 shows that the NP2 control of Botrytis bunch rot at harvest was more variable and less effective in the Sauvignon Blanc and Riesling grape varieties than in Chardonnay (Figure 2). In Season i, the NP2 treatment was no different from the Nil botryticide control, in preventing Sauvignon Blanc (SB) crop loss at vintage in Hawke’s Bay (Figure 3a), despite excellent control being obtained for Chardonnay (Figure 2c), when both varieties were grown simultaneously (same calendar year) under similar soil and climatic conditions on the same orchard.

Natural incidence of Botrytis rots at vintage and resulting crop losses, as shown in the Nil botryticides treatment, were higher in Hawke’s Bay than in Marlborough (Figure 3a,b). This reflects the climatic differences, where the moister climate in Hawke’s Bay is more favourable to Botrytis outbreaks. However, a significant reduction in SB crop loss with NP treatment was obtained in Hawke’s Bay versus Marlborough in Season ii (Figure 3b), which was thought to be related to the denser canopy management strategy adopted by Marlborough growers, and so the effect of canopy density was investigated in Season iii (Figure 3c).

**Figure 4:**
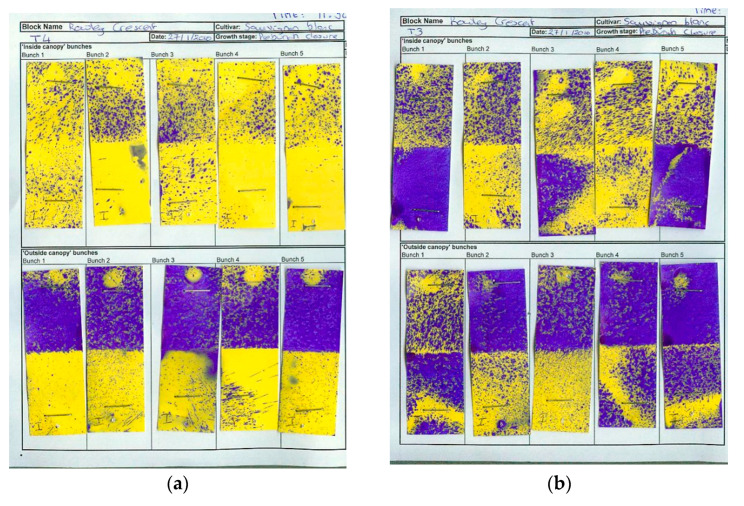
Moisture-sensitive papers indicating spray deposition in Marlborough, Season iii, under (**a**) standard canopy density (SCD), with approximately 26% bunch exposure; (**b**) low canopy density (LCD), with approximately 80% bunch exposure. The paper turns from yellow to purple when wet. The column of spray papers on the left-hand side in each picture indicates papers positioned on bunches on the outside of the canopy and the column on the right-hand side shows papers positioned on grape bunches inside the canopy.

**Figure 5:**
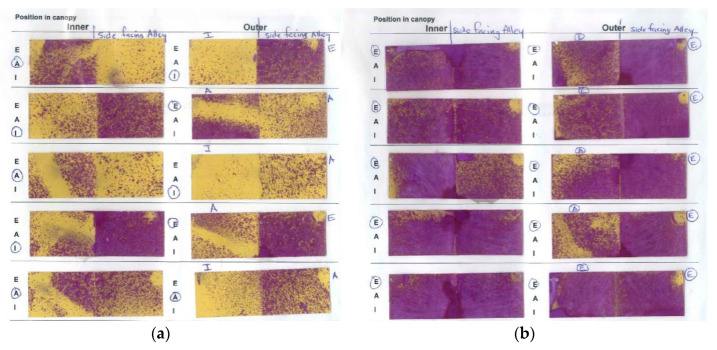
Moisture-sensitive papers indicating spray deposition in Hawke’s Bay, Season iii, under (**a**) standard canopy density (SCD, with approximately 26% bunch exposure); (**b**) low canopy density (LCD, with approximately 80% bunch exposure). The paper turns from yellow to purple when wet. The column of spray papers on the left-hand side in each picture indicates papers positioned on bunches on the inside of the canopy and the column on the right-hand side shows papers positioned on grape bunches on the outside the canopy. E = excellent spray coverage, A = adequate coverage, I = inadeqaute coverage, and the circled value represents the score given to that particular moisture-sensitive paper.

Irrespective of canopy density, NP2 application on SB grapes in Hawke’s Bay significantly reduced Botrytis crop loss, relative to the Nil botryticide control, and crop losses were also reduced by having a more open canopy (Figure 3c). In Marlborough, the natural levels of Botrytis were very low), but crop loss was still significantly reduced and control efficacy improved in the NP2, LDC versus the NP2, SCD treatment. This also correlated with better spray application efficiency in LCD than in SCD, especially to internal SB bunches, irrespective of trial site (Figure 4 and Figure 5), In general, there was greater spray penetration in both canopy densities in Hawke’s Bay than in Marlborough, and may have reflected slightly higher fruit exposure (resulting from heavier leaf plucking) at Hawke’s Bay (incomplete dataset not shown).

Efficacies in SB ranged from negative values (i.e., higher disease in the NP treatments than in the Nil botryticide control) to 96% efficacy (Figure 3).

Figure 3d shows that both NP1 (7 g/L) and NP2 (5–20 g/L) provided significant control of Botrytis in the field on Riesling vines, under a serious disease epidemic (up to 57% crop loss in untreated grapes). However, the NP treatments were not as effective as a full season treatment of different synthetic fungicides. There was no dose response of NP2 at the concentrations trialled, so a concentration as low as 5 g/L can be used effectively without loss of Botrytis disease control. NP1 had 46% efficacy and NP2 gave 48–58% efficacy.

### 2.2. PM Assessments

**Figure 6:**
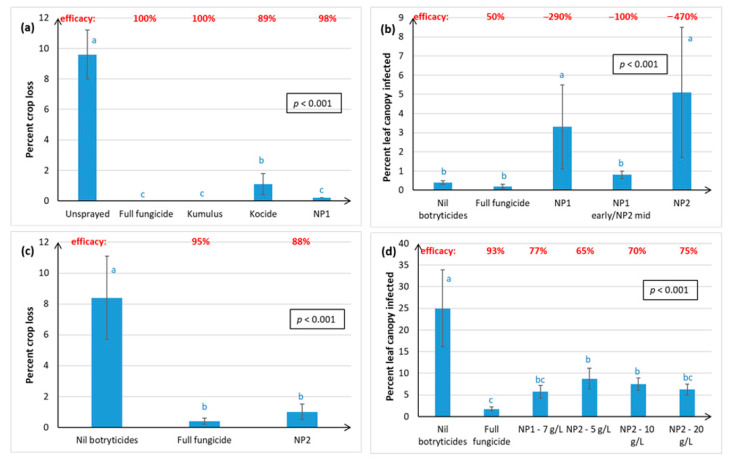
Percentage crop loss of fruit, or percentage leaf canopy infected by powdery mildew (PM) in Chardonnay and Riesling grape varieties. Crop loss was calculated as described in Figure 2, and efficacy as outlined in Figure 1. Percent canopy infected was the product of incidence (%leaves infected) × severity (%leaf area infected)/100. Treatments are described in full in Tables S1 and S2, and briefly in Figure 1 and Figure 2. Each graph represents data from a separate trial, set up in a randomised block resign, with error bars indicating standard errors, boxed values showing probabilities obtained from ANOVA, and different letters indicating statistically significant differences from pairwise likelihood ratio tests, which are only presented when the ANOVA *p* ≤ 0.05. The concentration of the active ingredient in the NP formulation is indicated in parentheses for each specific experiment. (**a**) Chardonnay, Season 1, Hawke’s Bay site, NP1 (7 g/L); (**b**) Chardonnay, Season 2, Hawke’s Bay site, NP1 (7 g/L) and NP (15 g/L); (**c**) Chardonnay, Season 3, Hawke’s Bay site, NP2 (5 g/L); (**d**) Riesling, Hawke’s Bay, NP1 (7 g/L) and NP2 (5, 10 and 20 g/L).

PM assessments were only carried out on some trials (Figure 6), and there was no assessment on Sauvignon Blanc grapes. This was either because no significant PM disease was observed during the course of the trial, or the commercial growers were unwilling to forgo PM control in their crop. However, even in the Nil botryticide treatment, where a minimum number of PM fungicides were applied to prevent exacerbated Botrytis infection, significant levels of PM infection could still sometimes be observed (Figure 6d).

PM data are presented in terms of percentage crop loss of berries resulting from PM infection (Figure 6a,c), and percentage infection of the leaf canopy (Figure 6b,d). In terms of PM disease control, NP1 performed on par with the full fungicide treatment in Chardonnay berries (Figure 6a), and in the Riesling leaf canopy (Figure 6d), but not in the Chardonnay leaf canopy, where disease control efficacy was worse than the Nil botryticide control. NP2 results were similar to NP1 (Figure 6b–d). Although single use of NP1 or NP2 during the mid-season produced negative efficacy values in the Chardonnay leaf canopy, using both NP1 and NP2 provided control as good as the Nil botryticides and full fungicide treatments (Figure 6b).

There was no dose response of NP2 at the concentrations trialled (5–20 g/L) on Riesling vines, so a concentration as low as 5 g/L can be used effectively without loss of PM disease control (Figure 6d).

Efficacy values on Chardonnay berries were 98% and 88% for NP1 and NP2, respectively (Figure 6a,c), and 77% and 65–75% on Riesling leaves (Figure 6d).

### 2.3. Fruit Maturity and Yield Assessments

Fruit maturity and yield measurements were not made in Riesling grapes, owing to budgetary restraints. 

#### 2.3.1. Fruit Maturity 

**Figure 7:**
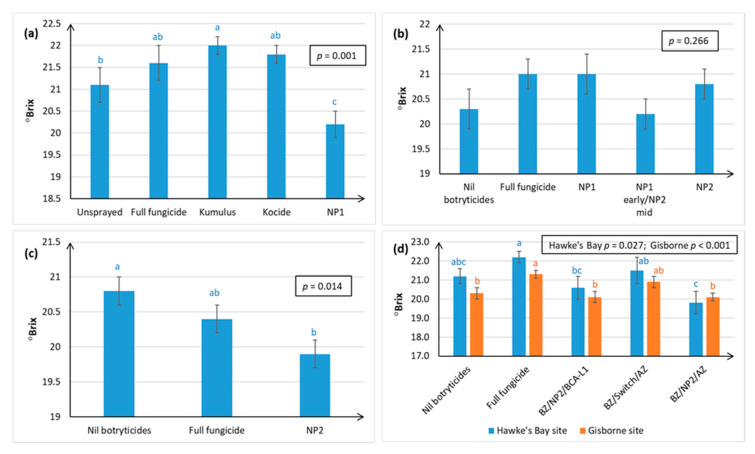
Fruit maturity (as indicated by °Brix) in Chardonnay berries, as assessed at harvest in April, over four successive seasons. Treatments are described in full in Tables S1 and S2, and briefly in Figure 1 and Figure 2. Each graph represents data from a separate season, with error bars indicating standard errors, boxed values showing probabilities obtained from ANOVA, and different letters indicating statistically significant differences from Fisher’s Least Significant Difference ((LSD, *p* ≤ 0.05), which are only presented when the ANOVA *p* ≤ 0.05. The concentration of the active ingredient in the NP formulation is indicated in parentheses for each specific experiment. (**a**) Season 1, Hawke’s Bay site, NP1 (7 g/L); (**b**) Season 2, Hawke’s Bay site, NP1 (7 g/L) and NP (15 g/L); (**c**) Season 3, Hawke’s Bay site, NP2 (5 g/L); (**d**) Season 4, Hawke’s Bay and Gisborne sites, NP2 (5 g/L).

Chardonnay fruit maturity at harvest (as indicated by °Brix) tended to be significantly lower in the NP1 treatment than all the other treatments when NP1 was applied throughout the growing season (Figure 7a), but that this problem could be mitigated by restricting use of NP1 and NP2 to the period between post-bloom and post-bunch closure (Figure 7b,c).

**Figure 8:**
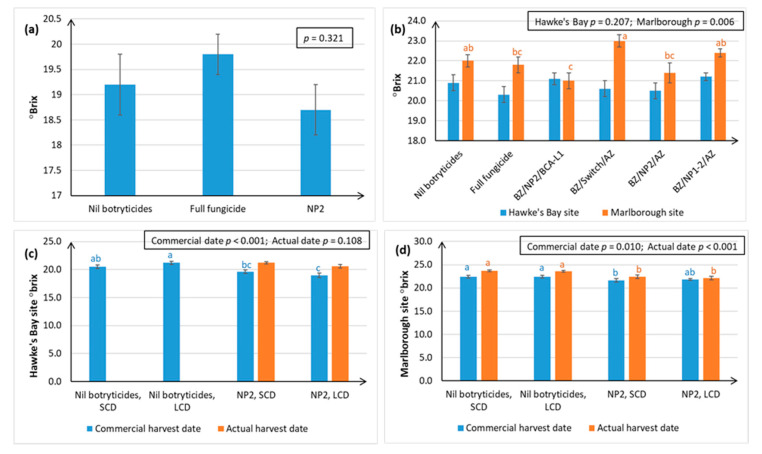
Fruit maturity (°Brix) in Sauvignon Blanc berries, assessed at harvest in April. Treatments are described in full in Tables S1 and S2, and briefly in Figure 1 and Figure 3. Error bars indicate standard errors, boxed values show ANOVA probabilities, and different letters indicate statistically significant differences from LSD (*p* ≤ 0.05), only presented when the ANOVA *p* ≤ 0.05. The concentration of the active ingredient in the NP formulation is indicated in parentheses for each specific experiment. (**a**) Season i, Hawke’s Bay site, NP2 (5 g/L); (**b**) Season ii, Hawke’s Bay and Marlborough sites, NP1 (5 g/L) and NP2 (5 g/L); (**c**) Season iii, Hawke’s Bay site, new formulation of NP2 (30 mL/L), °Brix were measured on two dates—the normal commercial harvest date and the actual harvest date, as berries were left on the vines for another week to allow for additional Botrytis development; (**d**) Season iii, Marlborough site, new formulation of NP2 (30 mL/L), Brix° measured on two dates. Data for the Nil botryticide treatments on the actual harvest date are missing in Figure 8c,d because the fruit were accidentally harvested by the commercial pickers.

A comparison between the Nil botryticide and NP treatments in Figure 8 shows that °Brix is not adversely affected in Sauvignon Blanc berries grown in Hawke’s Bay, when NP use is restricted to the period between post-bloom and post-bunch closure, and that °Brix is only slightly lower in the NP treatments in Sauvignon Blanc grapes grown in Marlborough.

The NP2 treatment had no significant effect on Sauvignon Blanc fruit maturity at harvest in Hawke’s Bay over three successive seasons (Figure 8a–c). In Marlborough, Season ii, there were differences in °Brix, between treatments, but these were not related to fungicide versus NP use (Figure 8b).

Irrespective of canopy density and trial site, °Brix tended to be lower in the NP2 treatments relative to the Nil botryticide controls, but the measurement of °Brix on two different harvest dates at each site shows that, whilst °Brix is retarded in the NP2 treatments, it continues to rise normally over time (Figure 8c,d). Canopy density did not have a significant effect on fruit maturity (Figure 8c,d).

#### 2.3.2. Yield Assessments

**Figure 9:**
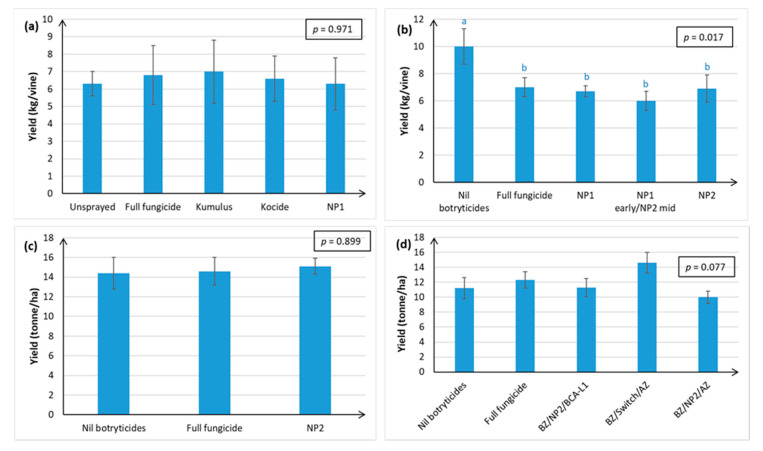
Fruit yield (kg/vine or tonne/ha) in Chardonnay grapes, as assessed at harvest in April, over four successive seasons. Treatments are described in full in Tables S1 and S2, and briefly in Figure 1 and Figure 2. Each graph represents data from a separate season, with error bars indicating standard errors, boxed values showing probabilities obtained from ANOVA, and different letters indicating statistically significant differences from Fisher’s Least Significant Difference ((LSD, *p* ≤ 0.05), which are only presented when the ANOVA *p* ≤ 0.05. The concentration of the active ingredient in the NP formulation is indicated in parentheses for each specific experiment. (**a**) Season 1, Hawke’s Bay site, NP1 (7 g/L); (**b**) Season 2, Hawke’s Bay site, NP1 (7 g/L) and NP (15 g/L); (**c**) Season 3, Hawke’s Bay site, NP2 (5 g/L); (**d**) Season 4, Hawke’s Bay site only (Gisborne site not measured at the discretion of the independent contractor performing the trial), NP2 (5 g/L).

Yield measurements for Chardonnay were completed over four successive seasons, but in Season 4 yield assessments were only made at the Hawke’s Bay site and not at the Gisborne site (at the discretion of the independent contractor who carried out the trial in Gisborne). Total yields in the NP1 (Figure 9a,b) and NP2 treatments (Figure 9b–d) were as good as the full fungicide control, regardless of whether yield was measured in kg/vine (Figure 9a,b) or tonne/ha (Figure 9c,d).

**Figure 10:**
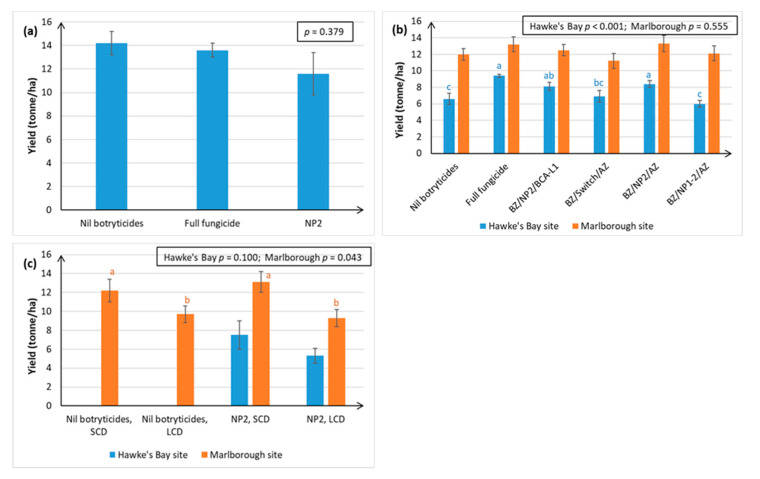
Fruit yield (kg/vine or tonne/ha) in Sauvignon Blanc grapes, as assessed at harvest in April, over three successive seasons. Treatments are described in full in Tables S1 and S2, and briefly in Figure 1 and Figure 3. Each graph represents data from a separate season. Error bars indicate standard errors, boxed values show ANOVA probabilities, and different letters indicate statistically significant differences from LSD (*p* ≤ 0.05), which are only presented when the ANOVA *p* ≤ 0.05. The concentration of the active ingredient in the NP formulation is indicated in parentheses for each specific experiment. (**a**) Season i, Hawke’s Bay site, NP2 (5 g/L); (**b**) Season ii, Hawke’s Bay and Marlborough sites, NP1 (5 g/L) and NP2 (5 g/L); (**c**) Season iii, Hawke’s Bay and Marlborough sites, new formulation of NP2 (30 mL/L). Data for the Nil botryticide treatments on the actual harvest date are missing in Figure 10c because the fruit were accidentally harvested by the commercial pickers.

Relative to the full fungicide treatment, the NP2 treatments (apart from use of both NP1 and NP2 during the mid-season) had no significant effect on Sauvignon Blanc yield over three consecutive seasons and at two different geographical sites (Figure 10). 

The single biggest factor affecting yield was canopy density, with LCD producing significantly lower yield than SCD in Marlborough (Figure 10c), possibly because there is less vegetative tissue to generate the carbohydrates needed for fruit development.

### 2.4. Effects of NPs on Wine Sensory Qualities and Residues (Season 2 Chardonnay Wines)

#### 2.4.1. Sensory Qualities

No sensory differences were detected between wines made from NP- and fungicide-treated Chardonnay vines (Season 2) (Table 1).

#### 2.4.2. Residue Analysis

The performance of the fatty acid methyl ester (FAME) analysis method was demonstrated by excellent recovery of a previously characterised flax seed oil that was spiked into a control treatment wine. The recovery of the trans-esterified fatty acids extracted from the flax seed oil was 92%. Similarly, the recovery of the internal recovery standard of heptadecanoic acid spiked into every sample before transesterification and extraction was excellent, providing values of 98 ± 10% (*n* = 10). The validated method provided a suitable level of sensitivity with a method detection limit (MDL) of 0.015–1 µg/mL for analysis of a 10 mL aliquot of wine or 0.5 mL of NP formulation product.

The FAME profiles of the formulated NP1 and NP2 products were dominated by a series of FAMEs of 16 (hexadecanoic acids) to 18 carbon chain lengths (octadecanoic acids) (Table 2). 

In contrast to the FAME profiles obtained for the NP formulations, those obtained for wines (Chardonnay, Season 2 vintage) prepared from treated grapes were characterised by a series of FAMEs of 4 (butanoic acid) to 12 carbon chain lengths (dodecanoic acid), with FAMEs of C-6 (hexanoic acid) and C-8 (octanoic acid) chain lengths dominating the profile (Table 2). The FAME profiles obtained from the NP-treated wines were further differentiated from those of the NP formulations by an absence of the series of C16 (hexadecanoic acids) to C18 (octadecanoic acids) FAMEs. In other words, the residues detected in the formulations are not present in the wines prepared from grapes treated with these formulations. Furthermore, the relative amounts of the FAMEs detected in the wines produced from grapes treated with the NP formulations were generally detected in considerably lower amounts than the FAMEs detected in the NP formulations. 

Moreover, comparison of the FAME profiles of the fungicide- and NP-treated wines showed no obvious differences (Table 2). In conclusion, analysis of FAMEs in wines produced from NP-treated grapes contain no detectable residues of the triglyceride tracer compounds characteristic of the NPs.

## 3. Discussion

In New Zealand, field conditions, natural product (NP) fungicides, NP1 (based on anhydrous milk fat) and NP2 (based on soybean oil), provided the effective control of Botrytis bunch rot on three green winegrape varieties (Chardonnay, Sauvignon Blanc, Riesling), and powdery mildew control on Chardonnay and Riesling, in two different geographical regions (Hawke’s Bay, Gisborne) over multiple seasons and under a full range of disease pressures. Disease control was usually as efficacious as the synthetic fungicide treatment(s) and total yields in NP-treated crops were normally equivalent to those obtained from the synthetic fungicide treatments. Microvinification of grape berries from the NP- and full fungicide treatments produced wines were evaluated by a trained sensory panel and subjected to chemical residue analysis. Results showed that the NP wines were residue-free and that there were no adverse effects on sensory characteristics. Taken together, the data indicate that disease control of two key pathogens in vineyards is possible using fungicides based on these natural products. 

The use of a plethora of naturally sourced products for plant disease control has been practised since ancient times and is often utilised by native peoples [17]. There is an abundance of global research on the use NPs under controlled laboratory conditions [17], but relatively fewer products have been developed for commercial use in variable field situations [15], often because of inadequate field research, problems associated with inconsistent efficacy, undesirable side effects such as growth of unwanted organisms, or difficulties with formulation [25]. Some of the most successful and widely used NPs that are commercially available include: sulphur, which remains the mainstay of powdery mildew control [26]; harpin proteins, which elicit plant defences [14]; chitosan, which is both an elicitor and directly antifungal [16]; giant knotweed extract (Regalia^®^) which is effective against PM [18]; seaweeds, although seaweed extracts are often used for growth enhancement rather than for disease control, per se [27]. Although plant oils are used frequently in horticulture as adjuvants [28], they are rarely the active ingredient in biofungicides, the exception being a tea tree oil extract (Timorex Gold^®^), which is effective against PM [29]. This study reports the novel use of an animal fat (NP1) and a plant oil (NP2) for the control of both PM and Botrytis bunch rot in a range of field trials, with data obtained on product efficacy over numerous seasons on different winegrape varieties, vineyards and geographic locations, and under different disease pressures. 

Both winegrape variety and geographical region appear to influence treatment efficacy against Botrytis. The NPs gave the most effective control of Botrytis rots on Chardonnay (ranging from 63–97% efficacy, as measured at harvest over four seasons), and much more variable control on Sauvignon Blanc (0–96% efficacy, measured over three seasons) and Riesling berries (46–58% efficacy, one season of data). Inter-variety variation existed even when trials were carried out at the same time, in the same vineyard, and using the same spray programme (e.g., Figure 2c versus Figure 3a), hence the grapevines would have been subjected to similar climatic conditions and vineyard management factors. Possible reasons for variations within the same vineyard, apart from inherent differences in resistance between the different varieties associated with microbiome, bunch architecture and berry biochemistry, might include the different ages of the vines and rootstocks (Table 3), along with possible variation in microclimate and soil type and microbiome within a particular vineyard. Whilst only results for green varieties are presented here, Calvo-Garrido et al. [30] independently tested an integrated disease management (IPM) programme containing the same products as our BZ/NP2/AZ treatment on red grape varieties in France and found it to be the most effective for Botrytis control out of all the biological programmes tested. In general, Botrytis disease control was much more effective in Hawke’s Bay and Gisborne than in the dryer climate of Marlborough, which is less conducive to Botrytis infection (Figure 2 and Figure 3). Regional differences may also be attributable to subtle differences in *B. cinerea* ecology and epidemiology [5], climate, soils, rootstock, and vine age, as well the effect of vineyard management practices. For example, Marlborough growers normally maintain denser Sauvignon Blanc canopies (≤40% bunch exposure) than Hawke’s Bay growers (typically ≥70% bunch exposure) to create the grassy flavours that are preferred in wines from this region. The effect of canopy density on NP spray penetration and Botrytis disease control efficacy was investigated in the current study. Spray penetration of the non-systemic NPs was much more effective in the low-density canopy (LCD, approximately 80% bunch exposure) versus the standard density canopy (SCD, approximately 26% bunch exposure) at both sites. Disease control efficacy results in Marlborough were inconclusive, due to very low levels of Botrytis, but the Hawke’s Bay results indicated that a more open canopy helps to reduce Botrytis in its own right, and that canopy density also has a significant effect on the efficacy of the NPs (Section 2.1.2). An LCD allows for greater air circulation around the bunches, thus lowering humidity, and creating a micro-environment that is less favourable to Botrytis [31]. However, the most likely reason for NP2 working better in an LCD is that it enables greater spray penetration to internal bunches (Figure 3 and Figure 4), which is important to NP2 efficacy, because it has contact-only activity [23,32]. However, yield tended to be significantly lower in the LCD, possibly because insufficient leaf material remained to create adequate photosynthates for berry development. Further work is, therefore, required to determine the optimal canopy density for excellent disease control without adverse effects on yield. Another possibility might be plucking just one side of the canopy, as was successfully used in the study by Calvo-Garrido et al. [30]

An important aspect of the efficacy of the NPs against Botrytis is that they can inhibit latent infections (Section 2.1.1), which generally occur around the mid-season (i.e., during the summer months) of the phenological stages of grape development. Switch® is the main (systemic) fungicide used to provide control against Botrytis latent infections, but its use is restricted to two applications/season and the final application date should be no less than 60 days before harvest to prevent maximum residue limits being exceeded in export wines and to the minimise the risk of developing fungicide resistance [33]. Use of the NPs offers a viable residue-free alternative (Section 2.4.2) for latent infection control. However, it must be noted that the NP1 and NP2 residue studies were carried out on wines produced from Chardonnay grapes in the Hawke’s Bay region. We recommend that further residue studies are performed on wines produced from different varieties and regions to confirm this result. Another advantage of the NPs is that pathogens do not tend to develop resistance to agricultural sprays containing lipids [19,20]. Given that NPs, like other lipids, appear to have contact-only action via a direct physical effect on the pathogen [23,32], control of latent infections is surprising and may suggest an, as yet undiscovered, mode of action, such as the direct induction of plant defences, or possibly indirect induction of defences via generation of fungal elicitors (compounds that activate plant defences) following the disruption of pathogen cells. This remains to be investigated.

The efficacy of the control of PM on berries was only recorded on Chardonnay (88–98% control, two seasons of data) and Riesling (65–77% control, one season) in the Hawke’s Bay. PM was either not measured or not observed in Sauvignon Blanc in the current study. However, approximately 40% control of PM on Sauvignon Blanc grapes was achieved with use of NP2 in an LCD in Marlborough in another study (Wurms, PFR, pers. comm.). The reasons for varietal and geographical variations are likely to be the same as discussed for Botrytis, but there is an additional complication. The teleomorphic stage of *Eryspihe necator*, a key source of inoculum for the next season, is becoming much more prevalent in commercial New Zealand vineyards [11] and is directly associated with increased disease severity [12]. At this stage, the relative efficacy of the NPs against the teleomorphic stage of PM is unknown, and this needs further research. More information is also needed on population structure and the distribution of mating types in New Zealand.

The BZ/NP2/AZ programme represents a successful season-long biopesticide programme using products that are commercially available in New Zealand and comprising one biocontrol agent (BCA) and two different natural products, all of which have different modes of action. The combined use of the three products is likely to increase the durability of the individual components compared with the durability if they were used alone. The efficacy of this treatment programme has recently been confirmed in France [30]. The fungus (*Ulocladium oudemansii*) in BOTRY-Zen works by outcompeting *B. cinerea* for the colonisation of floral tissue and bunch trash before bunch closure, thus preventing *B. cinerea* infections from becoming established within the bunches [34]. NP2, which has been registered in New Zealand under the trade name MIDI-Zen^®^, directly affects PM and Botrytis pathogens by causing conidiophores to collapse and conidia and hyphae to wither and extrude cellular contents. ARMOUR-Zen^®^ contains chitosan, which is known to be both directly fungicidal and to induce plant defence mechanisms [35,36]. The BZ/NP2/AZ treatment is also known to be residue-free (Section 2.4.2), which represents a major advantage of using NP2 as an alternative to Switch, and does not adversely affect wine sensory quality (Section 2.4.1), or yield (Section 2.3.2). However, it can cause a delay in °Brix when compared to BZ/Switch/AZ (Figure 8b). 

There is often a delicate balance between disease control efficacy and higher doses of oils/fats becoming phytotoxic to the plant, as fats and oils are often associated with chlorosis and necrosis of plant tissue [37,38,39,40], so this may explain why higher doses of NPs are not always beneficial (Figure 3d). Combining the two NPs together has been shown in other studies to have a complementary effect on disease control, making it possible to reduce the concentrations of the active ingredients (a.i.) of each [25], possibly because the NPs appear to have different physical modes of action, with NP1 causing withering and distortion/ridging of fungal structures, whilst NP2 application leads to the extrusion of cellular contents [23]. However, in this study, using alternating NP1 and NP2 at different times in the spray programme, instead of the use of just one NP product, seemed to exacerbate Botrytis latent infections (Figure 1a). Significant phytotoxic effects associated with use of the NPs, such as leaf burning, were not observed at any time in the current study.

As with all agricultural products, the successful deployment of NPs depends on understanding their potential drawbacks. Accurate spray targeting, particularly under dense canopies, remains the most critical factor in the success of these products, because the NPs appear to have contact-only action. The effect of NPs on fruit maturity also needs to be considered, as °Brix (which is used an indicator of fruit maturity in grape berries) was often 1–1.5° lower at harvest time. Application of lipids over an extended period of time can be associated with a delay in the onset of véraison [41,42], hence this problem was minimised by instead restricting their use to the mid-season as part of an IPM programme. Figure 8 also shows that °Brix continues to rise normally over time in the NP treatments, so, if necessary, harvest can be delayed by 1–2 weeks to achieve optimal °Brix. However, further research is also needed to determine whether the reduction in acids in the berries is also delayed. If acid reduction is not delayed, then the fruit would need to be picked at a lower °Brix, in which case NP-use might be another tool to produce lower alcohol wines harvested at lower °Brix. 

Overall, this research has shown that the effective control of PM and Botrytis can be achieved in winegrape vineyards using natural lipid-based products, without any adverse effects on yields, and offering the additional advantage of being residue free, not easily overcome by pathogen resistance, and consisting of ingredients that are generally regarded as safe. 

## 4. Materials and Methods

### 4.1. Trial Sites

Field trials were performed on a number of different research blocks and commercial vineyards on different varieties, at different geographical sites and over several seasons, as summarised in Table 3.

### 4.2. Spray Treatments and NP Formulations

Spray applications in these trials were at 3- to 21-day intervals (typically 14 days), from the phenological stage of flowering up until two weeks before vintage. Before bloom, all vines received grower-applied standard fungicides for disease control, especially for control of PM resulting from germination of overwintering conidia in flag shoots. In some trials the main focus was on Botrytis rather than PM control, and since Botrytis can be exacerbated in PM-infected plants, the “no-fungicide” controls were actually “nil botryticide” controls and included fungicides for the control of PM. Full details of the spray schedules are provided in Supplementary Tables S1 and S2. 

PFR’s Bioprotia™-(Hamilton, New Zealand) series control products NP1 and NP2 were normally prepared as 25- to 35-fold emulsifiable concentrates (ECs), and diluted with water to field application rates immediately before use. The EC formulations comprised three ingredients: the natural product active ingredient (a.i.)—anhydrous milk fat for NP1, or soybean oil for NP2; a food-grade emulsifier to create the oil in water emulsion; a food-grade antioxidant to prevent oxidative degradation of long chain fatty acids and the associated development of rancid odours. The full identity of the NP formulations is commercially sensitive, but common co-ingredients are outlined in the patent (Section 6) and by Wurms and Ah Chee [43], and the concentration of the a.i., which is only part of the formulation that is directly anti-fungal (Section 6), is always specified. The ECs were stored at room temperature in a secure shed along with other agrichemicals when not in use. Optimal application concentrations of the active ingredients were determined by trial work as 5–15 g/L for both NP1 [25] and NP2 (Section 2). In the early trials, the NPs were applied for the full season, whereas in later trials the NPs were included as part of an integrated biopesticide spray programme, as this was deemed to provide multiple modes of action and thus be more durable than using just one product alone. For the integrated spray programme, BOTRY-Zen (Dunedin, New Zealand), 4 kg/ha formulation of the biocontrol agent (BCA), *Ulocladium oudemansii*) was applied early-season from 5% capfall to post bloom, NP1 and/or NP2 application was during the mid-season from berries pea-size to post-bunch closure, and BCA-L1, an experimental formulation of *Aureobasidium pullulans* BCA (2 × 10^7^ spores/mL) was applied during the late-season from véraison to two weeks pre-vintage. 

The full-season fungicide spray schedule and the water rate per ha (typically 500 L–1000 L/ha) was determined in accordance with New Zealand wine industry recommendations to members at the time (www.nzwine.com).

### 4.3. Botrytis Assessments

All field trials allowed for natural infection, i.e., there was no artificial inoculation.

#### 4.3.1. Latent Infections

A sample size of 50 berries/plot was assessed for latent *B. cinerea* infections using the Overnight Freezing and Incubation Technique (ONFIT) [44]. This method eliminates all micro-organisms from the fruit surface, then kills immature fruit tissues by overnight freezing, thus allowing latent micro-organisms, such as *B. cinerea,* within the immature fruit tissue to re-commence growth when incubated in favourable conditions. Berries for ONFIT were first surface sterilised by dipping in 95% ethanol for 30 s, then 0.5% sodium hypochlorite in 0.5% Tween^®^ 80 for 5 min, and 95% ethanol again for 30 s, then rinsed in running tap water. They were then placed onto plastic grids and frozen at −18°C for 24 h, then incubated at 15°C in the dark. The number of berries developing infection after 10–12 days was expressed as a percentage.

#### 4.3.2. Infection at Vintage

For Botrytis assessment of berries at vintage, 50 bunches per plot were inspected for incidence (% of bunches infected) and severity (% of bunch area covered by Botrytis). Percentage crop loss caused by Botrytis, assessed at harvest, was calculated using the formula (%bunch severity × %bunch incidence)/100 for 50 randomly harvested bunches per plot. To allow the comparison of the performance of spray programmes between years, or between trials, the percentage disease reduction (efficacy) for each programme was calculated and expressed as a percentage of the disease in the Nil botryticide treatment. The formula for efficacy is: E = ((U − T)/U) × 100, where E is the efficacy of the programme, T is the percentage disease (incidence or crop loss) for the programme and U is the percentage disease in the Nil botryticide treatment (i.e., untreated) [45].

### 4.4. PM Assessments

PM infections also developed naturally, i.e., no artificial inoculation. For PM assessments on fruit, 50 bunches per plot at vintage were inspected for incidence and severity, with percentage crop loss calculated as the product of these two values. For canopy assessments, 50 randomly selected leaves were assessed per plot. PM assessments were not made in any of the Sauvignon Blanc trials because disease levels were negligible.

### 4.5. Fruit Maturity and Yield Assessments

Degrees Brix (°Brix, percent soluble solids, which are largely sugars) was used as a measure of fruit maturity and was evaluated at vintage on juice extracted from a randomly selected 50-berry sample/vine using an Atago^®^ digital refractometer (Atago Co. Ltd., Tokyo, Japan).

Fruit yield was assessed by harvesting individual treatment vines at vintage to obtain total kg fruit/vine and the number of bunches per vine. Individual yield components (bunch and berry numbers and weights) were measured by harvesting all bunches within a designated zone for each plot. 

### 4.6. Experimental Design and Statistical Analysis

Unless otherwise specified, all experiments were set out as complete randomised block designs. All non-percentage data were subjected to Analysis of Variance (ANOVA), with replicate and treatment as factors. For percentage data, binomial generalized linear models were used, to avoid the need for data transformation. For these linear models the number of observations was assumed to be 100, with a dispersion factor estimated from the residual deviance. In cases where there was a significant treatment effect, mean separation (*p* ≤ 0.05) was carried out using Fisher’s Least Significant Difference (LSD) for ANOVA and by pairwise likelihood ration tests (*p* ≤ 0.05) for the binomial generalized linear models, using GenStat Release 20 software (VSN International Limited, Hemel Hempstead, UK).

### 4.7. Individual Trial Details

Research was carried out in collaboration with the New Zealand wine industry with the aim of obtaining season-long disease control of Botrytis and PM using soft fungicides, such as the NPs, within an IPM system. Changes were made from season to season to ensure the continual optimization of the system by better understanding the effects of grape variety, geography and associated climate, disease pressure, rate and frequency of product application, etc. Slight variations to protocols between the various seasons are outlined in this section. 

#### 4.7.1. Chardonnay—Season 1

This represented the first test of NP1 efficacy in a vineyard. Chardonnay grapevines (*Vitis vinifera*) were sited on a research block (vineyard A, Table 3) in Hawke’s Bay, New Zealand.

Nine spray applications of the treatments (Table S1) were applied between capfall (mid-November) and 2.5 weeks before harvest (mid-March) at 8- to 21-day intervals, using a motorised, moderate pressure handgun, at an application rate of 500 L/ha during capfall and thereafter at 800 L/ha. An emulsifiable concentrate of NP1 was diluted 35 fold with water immediately prior to use to produce an application rate of 7 g/L. 

Percentage crop loss caused by Botrytis was assessed at vintage (early April), and PM infections on fruit were assessed at véraison (mid-February). °Brix and yield were measured at vintage (April).

There were four replicate vines per treatment, with each replicate vine in a separate row (plot). Each treatment plot consisted of three vines in a single row with the two edge vines acting as buffer vines to prevent the cross-contamination of disease and over spray. The actual treated plot was 6 m long, being equivalent to 15 m^2^ of vineyard area. 

#### 4.7.2. Chardonnay—Season 2

In this season, a second NP (NP2) was also evaluated in the field. Vineyard B (Table 3) in Hawke’s Bay was used for this trial. Fruiting zone leaf removal, to optimise fungicide deposition and to enhance berry quality, resulted in approximately 85% bunch exposure and was carried out just before the second bloom spray (80% capfall) and again before véraison. 

Treatments (Table S1) were applied using a motorised moderate-pressure handgun at a water rate of 500 L/ha during capfall and thereafter at 750 L/ha. Unlike in season 1, where NP1 was applied from capfall (November) to 2.5 weeks before harvest (March), NP applications in this trial were restricted to one spray each at post bloom (mid-December), pre-bunch closure (early-January) and post-bunch closure (late-January), i.e., predominantly during the mid-season of grape phenological development. There were two reasons for this—firstly, the NPs were applied as part of an integrated season-long biological spray programme, because using products with different modes of action is more durable, and secondly, the use of NPs was avoided after véraison to minimise the delay in °Brix. 

Latent *B. cinerea* infections were assessed pre- and post-bunch closure, using ONFIT. Percentage crop loss from Botrytis bunch rot, fruit maturity, and crop yield were measured at vintage (early-April) and PM canopy assessments were carried out on approximately 50 randomly selected leaves per plot, 10 days after vintage (mid-April).

Each treatment plot was a vineyard bay (7 m in length) consisting of two large vines, with the first and last metre of each plot being used as a buffer zone. There were five replicate plots for each treatment. 

#### 4.7.3. Chardonnay—Season 3

Chardonnay vines from a commercial block in vineyard C (Table 3) were used for this trial, with NP2 rather than NP1 being the focus, because preliminary trials suggested better control of Botrytis latent infection by NP2 (K. Wurms, PFR, pers. comm.). The field application rate of NP2 was also reduced from 15 to 5 g/L.

Leaf removal in the fruiting zone by manual plucking took place just prior to the second bloom spray and again before the véraison, achieving on average 75% fruit exposure. 

All treatments (Table S1) were applied at 3- to 19-day intervals from 5% capfall (mid-November) through to 2 weeks pre-vintage (late-March), using a motorised, moderate-pressure (150 psi), high-volume handgun at an application rate of 500 L/ha. Applications were always applied at least six hours before rainfall. 

Just prior to application of sprays at véraison (early-February), latent *B. cinerea* infections were assessed using ONFIT. At vintage (early-April), Botrytis bunch rot and PM crop loss, spray efficacy, fruit maturity, and yield parameters were measured/calculated. 

There were five plots/treatment, with each plot consisting of one vineyard bay (9 m of canopy).

#### 4.7.4. Chardonnay—Season 4

The effect of using different components in the NP-IPM programme was investigated, along with determining whether the same results could be obtained in different regions. Research blocks were used at two different geographical sites in New Zealand—Hawke’s Bay (Vineyard B, Table 3) and Gisborne (Vineyard D, Table 3). Gisborne tends to be warmer and more humid later in the season than Hawke’s Bay, so is very prone to Botrytis. At Vineyard B, fruiting zone leaf removal resulted in (on average) 85% bunch exposure and was carried out just before the second bloom spray (80% capfall) and again before the pre-bunch closure spray. To control post bloom PM, Topas 200EW (12.5 mL/100 L) was applied in later-December to all treatments but the NP2 plots. Additional Topas sprays were required over the whole trial on mid- and late-January because of the high-risk PM season. Treatments (Table S1) were applied using a motorised moderate-pressure handgun at a 500 L/ha water rate. Each treatment plot was a vineyard bay (7 m in length) consisting of two large vines, with the first and last metre of each plot being used as an intra-plot buffer zone. Bare land existed at both sides of the trial area, so side buffer plots were not required. There were six replicate plots for each treatment, with a buffer plot at each end of the trial area.

The Gisborne trial at Vineyard D was carried out by an independent contractor. To control post bloom PM, Kumulus (3 kg/ha) was applied mid-season (late-December to late-January). Trial applications commenced at early- to mid-flowering (late-November) and were applied with a Solo 450 mist blower (airshear nozzle) at 750 L per hectare. Plots were 7.2 m long and contained four vines, with the first and last vine of each plot being used as a between-plot buffer zone. There were six replicates per treatment, with each replicate in a separate vineyard row with two buffer plots at the northern end of the trial area. There was a headland on the southern end which formed the vineyard boundary. Buffer plots were sprayed with botryticides with the mist blower at the same time as the trial area.

Collection of data was the same as those described for Season 3, with the exclusion of PM data.

#### 4.7.5. Sauvignon Blanc—Season i

A parallel trial to the one carried out on Chardonnay (Season 3) was carried out on the same vineyard at the same time on Sauvignon Blanc grapes to compare the efficacy of spray programmes on these two varieties grown under common soil and environmental conditions. There were five replicate plots (5 m in length, containing 2.5 vines) per treatment. Leaf plucking to attain fruit exposure (75% exposure on average), application of treatments (Table S2), assessments of Botrytis latents, harvest disease assessments, measurement of yield parameters and data analysis were the same as described for Chardonnay, Season 3.

#### 4.7.6. Sauvignon Blanc—Season ii

To investigate whether the same results could be obtained in different regions for the Sauvignon Blanc variety, field trials were established in commercial vineyards in two geographic regions in New Zealand—Marlborough and Hawke’s Bay. The climate during summer in Marlborough is often dry so Botrytis does not tend to be as problematic as in Hawke’s Bay. 

Vineyard E (Table 3) was used as the Hawke’s Bay site. Fruiting zone leaf, resulted in (on average) 80% bunch exposure, and was carried out just before the second bloom spray (80% capfall) in mid-December, and again prior to the pre bunch closure (PBC) application in early-January. To control post-bloom PM, Topas 200 EW (1.25 mL/L) was applied in late December to all but the NP2 treated areas. Because of a high-PM-risk season, additional Topas sprays were required over the whole trial during mid- and late-January. Treatments (Table S2) were applied using a motorised moderate-pressure handgun, at 500 L/ha. Each treatment plot was a vineyard bay (5.6 m in length) consisting of four vines. Each replicate was in a separate vineyard row, with a buffer row of grapes on either side of the trial area and a buffer plot (vineyard bay) at each end of the trial area. There were six replicate plots for each treatment.

Marlborough vineyard F (Table 3) was the only vineyard in this work that was situated in the South Island of New Zealand. Removal of leaves around the fruiting zone to optimise fungicide deposition resulted in c. 80% bunch exposure and was carried out twice by machine using a Gregoire leaf plucker in mid-January and late-February, and once by hand in early-February. Treatments were applied using a handgun, powered by a 12-volt pump, supplied from a 240 L trailer sprayer, towed by a quad bike. A water rate of 600 L/ha was applied throughout the season. To control post-bloom PM, Topas was applied on late-December to all but the NP2-treated vines. Each treatment plot was a vineyard bay (7.2 m long), consisting of four vines in a single row, with the first half of the vines at each end of every plot being used as a buffer zone. There were eight replicate plots for each treatment, laid out in a randomised complete block design. Each replicate was in a separate vineyard row with a buffer row of grapes on either side of the trial area and two buffer plots at each end of the trial area. Buffer plots were sprayed with botryticides by handgun at the same time as the trial area.

For both trial sites, assessments of Botrytis latent infections, harvest disease assessments, measurements of yield parameters and data analyses were the same as described in Section 4.7.3.

#### 4.7.7. Sauvignon Blanc—Season iii

Field trials were established in Sauvignon Blanc in Hawke’s Bay and Marlborough to further investigate regional differences, and also the effect of canopy density on Botrytis control. This is because Marlborough growers normally maintain denser canopies (≤40% bunch exposure) than Hawke’s Bay growers (typically ≥70% bunch exposure) to create the grassy flavours that are preferred in Sauvignon Blanc wines from this region. Relative to all the previous trials, the formulation included a different antioxidant, because the antioxidant used previously had been taken off the market. To compensate for the new formulation, a higher-than-normal field application rate of the a.i. was used, i.e., 30 mL/L of NP2.

In the Hawke’s Bay research vineyard E (Table 3), leaf plucking and shoot thinning procedures were applied to create c. 80% bunch exposure in the low (optimised) canopy density (LCD) treatments, and c. 26% bunch exposure in the standard canopy density (SCD) treatments. To control post-bloom PM, Topas 200EW (1.25 mL/L) was applied over the whole trial in late-December and again in early-February. The trial treatments (Table S2) were applied using a 15-L knapsack sprayer, at 500 L/ha. Actiwett^®^ was applied at 0.25 mL/L as an adjuvant to all products and contained 980 g/L linear alcohol ethoxylate. Each treatment plot was a vineyard bay (5.6 m in length) consisting of four vines, with the first and last vine of each plot being used as a between-plot buffer zone. The replicates were sequentially laid out across the vineyard rows to account for a slight gradient in disease risk down the row. The trial was buffered with a row of grapes on either side of the trial area and a buffer plot (vineyard bay) at each end of the trial area. There were six replicate plots for each treatment, laid out in a randomised complete block design. 

Creation of LCD and SCD in Marlborough research vineyard G (Table 3), and application of sprays were the same as described for Hawke’s Bay. To control post-bloom PM, Topas was applied in early January to all treatments. Each treatment plot was a vineyard bay (7.2 m long), consisting of four vines in a single row, with the first half of the vine at each end of every plot being used as a buffer zone. There were six replicate plots for each treatment, laid out in a randomised complete block design. Each replicate was in a separate vineyard row.

At both sites, assessments of Botrytis latent infections, harvest disease assessments and measurement of yield parameters were the same as described in Section 4.7.3. An indication of spray penetration was obtaining by placing moisture-sensitive spray papers in the plucked and unplucked canopies in mid-January in Hawke’s Bay, and late-January in Marlborough. However, owing to low levels of Botrytis development at the Marlborough site, grapes were left on the vines at both sites for an additional week later than the commercial harvest dates, to allow for additional Botrytis development. Harvest maturity was sampled at both the commercial harvest dates and the actual harvest dates, and this allowed us to look at changes in °Brix over a week. 

#### 4.7.8. Riesling Trial

The trial on the Riesling variety was one on the earliest trials and was performed to determine the best field application rate for NP2. Riesling vines were located on commercial block in vineyard B (Table 3), with treatments shown in Table S2. Fruit zone leaf removal to optimise fungicide deposition, resulting in 85% bunch exposure, was carried out just before the second bloom spray and before véraison. Each treatment plot consisted of half a bay (two vines) and there were four replicate plots/treatment, laid out in a complete randomised design (CRD). Spray application was the same as described in Section 4.7.2, except that a 600 L/ha water rate was used. One week post capfall, all vines received a single application of Topas^®^ 200 EW (12.5 mL/100 L) for PM control. Percentage crop loss caused by Botrytis and PM was assessed at vintage (early-April). Owing to budget constraints, yield and maturity parameters were not measured.

### 4.8. Sensory Qualities

Wines were made from Chardonnay Season 2 grapes from vineyard B (Table 3) and were bottled in 750 mL Bordeaux bottles under screw cap. Microvinification took place at the Eastern Institute of Technology (EIT), Taradale, New Zealand.

For sensory analysis, each variety was analysed separately by a trained sensory panel of five wine judges. The bottles were set out with the different Chardonnay vintages × treatments randomly distributed. The wines were all opened and poured at the same time and evaluated in one flight. Standard ISO Wine Tasting glasses were used.

The wine’s appearance and nose were numerically scored as well as commented on. The palate and overall quality of the wine was not scored numerically but commented on to the extent that it was either "faulty" or "not faulty", on the palate. Faults were further differentiated to be either winemaking fault/s, e.g., oxidation occurring during the winemaking process or non-winemaking fault/s. Flavour taints and appropriate descriptors of the taints were noted, if present.

### 4.9. Residue Analysis

A literature review of the main chemical constituents present in NP1 and NP2 was conducted to identify classes of compounds amenable for use as chemical tracers of residues in finished wines, and identified triglyceride lipids as ideal tracers. Triglycerides are readily trans-esterified to their respective fatty acid methyl esters (FAMEs), which can be analysed by high-resolution capillary gas chromatography (GC) or gas chromatography mass-spectrometry (GCMS). 

**Figure 11:**
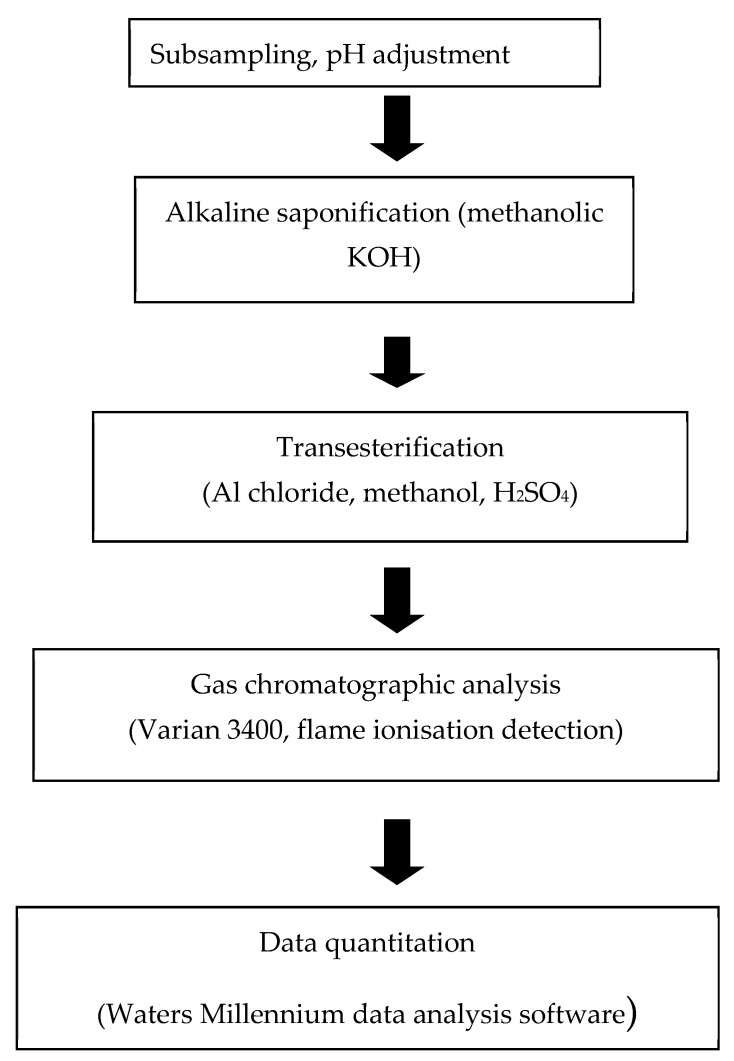
Schematic summary diagram of fatty acid methyl esters (FAMEs) analysis method.

Formulated NP1 (7 g/L) and NP2 (20 g/L) product, and finished wine prepared from corresponding treatment plots (Chardonnay Season 2 grapes from vineyard B) were selected for validation of the triglyceride/FAME residue analysis method. Wines were made from treatments that had received the highest application rates of NPs to grapes in the trial plots, and, therefore, represented the worst-case scenario for the potential carryover of NP derived residues into the finished wines. The FAMEs extraction procedure is summarised in Figure 11. 

## 5. Conclusions

Overall, this research demonstrates consistent results over numerous seasons showing that lipid-based natural product fungicides achieve effective control of Botrytis and PM on field-grown winegrapes on a par with synthetic fungicides. In addition, these biofungicides are residue free, have no adverse effects on yield, contain food-extract-based constituents that have low toxicity and are environmentally benign, and have been successfully integrated with the use of other biocontrols. However, activity is contact-only, requiring excellent spray penetration in dense crops, and the use of oils can delay fruit maturity and, therefore, harvest by up to 1–2 weeks. 

## 6. Patents

A patent has been published from this work: Wurms K, Ah Chee A. 2006. Fungicidal Compositions, Patent No WO2006006878. The patent (full for NP1 and divisional for NP2) has been granted in New Zealand, Australia, Chile, the United States of America, France, Germany, Italy, the Netherlands, Poland, Portugal, Spain, Turkey and the United Kingdom. A trademark (Bioprotia™) has also been applied to bioprotection products developed by Plant & Food Research, covering NP1, NP2 and other biological control products. 

## Figures and Tables

**Table 1:** Sensory analysis (by a trained sensory panel of five wine judges) of Chardonnay wines made from the Season 2 vintage from Hawke’s Bay.

Treatment Code	Wine Quality	Wine Making Fault	Colour	Aroma Score	Aroma/Palate Fault	°Brix
NP1 Full Season	Not faulty	None	Pale green/neutral	9	None	21.5
NP2 Full Season	Not faulty	None	Pale green/neutral	9	None	21.0
Fungicide Full Season	Not faulty	None	Pale green/neutral	8.5	None	23.2

**Table 2:** Peak area response of fatty acid methyl esters (FAMEs) identified in Natural Product (NP) formulations (Formula) and treated finished wine samples (Wine) from the Chardonnay Season 2 vintage from Hawke’s Bay. Flax seed oil was spiked into the control (fungicide) treatment wine to test for recovery of trans-esterified fatty acids.

FAMEs	Wine—Fungicide Treated	Wine—NP1 Treated	Wine—NP2 Treated	Wine—Fungicide + Spike	Formula—NP1	Formula—NP2
Butyric, C4:0	9491	10,821	9443	7272	119,350	
Hexanoic, C6:0	66,545	73,284	69,557	56,837	117,300	
Octanoic, C8:0	138,341	155,877	146,758	132,519	69,840	
Decanoic, C10:0	39,887	41,987	43,831	39,427	171,530	
Dodecanoic, C12:0	453	547	425	540	203,440	
9-tetradecanoicacid, C14:1 (myristoleic)					54,340	
Tetradecanoic, C14:0					752,170	9760
Pentadecanoic, C15:0					79,520	
9-hexadecanoic acid, C16:1(palmitoleic)					89,240	10,940
Hexadecanoic, C16:0				22,442	2,561,290	1,438,840
Heptadecanoic, C17:0, (IS) **^1^**	186,389	201,247	202,954	211,386	220,450	160,000
*cis*-9-*cis*-12-octadecanoic acid, C18:2 (linoleic)				57,968	1,692,320	7,021,880
α-linolenic acid, C18:3 (linolenic)				272,564		725,670
*trans*-9-trans-12-octadecanoic acid, C18:2 (linolelaidic)				59,467	2,048,930	3,743,800
*cis*-9-octadecanoic acid, C18:1 (oleic)				5317	233,340	534,890
*trans*-9-octadecanoic acid C18:1 (elaidic)					376,170	84,720
Octadecanoic, C18:0 (steric)					137,750	15,030

^1^ IS = internal recovery standard.

**Table 3:** Vineyard trial sites.

Vineyard ID	Location	Variety Clone/Rootstock/Vine Age (years)	Vine Spacing—Row by Vine (m)	Vines/ha	Season Used ^1^
A	Hawke’s Bay	Chardonnay/UCD6/3309/11 years	2.5 × 2.3	1739	Season 1
B	Hawke’s Bay	Chardonnay/UCD15/3309/7 years	2.75 × 3.5	1039	Season 2
C	Hawke’s Bay	Chardonnay/UCD5/SO4/10 years	3.1 × 1.8	1792	Season 3
B	Hawke’s Bay	Chardonnay/UCD15/3309/9 years	2.75 × 3.5	1039	Season 4
D	Gisborne	Chardonnay/Clone95/Schwarzmann/10 years	3.0 × 1.8	1852	Season 4
C	Hawke’s Bay	Sauvignon Blanc/mass-selected^2^/SO_4_/7 years	3.1 × 2.0	1613	Season i
E	Hawke’s Bay	Sauvignon Blanc/mass-selected^2^/SO_4_/7 years	3.0 × 1.4	2381	Season i
E	Hawke’s Bay	Sauvignon Blanc/mass-selected^2^/SO_4_/8 years	3.0 × 1.4	2381	Season ii
F	Marlborough	Sauvignon Blanc/BDX 317/101-14/11 years	2.7 × 1.8	2058	Season ii
E	Hawke’s Bay	Sauvignon Blanc/mass-selected^2^/101-14/20 years	3.0 × 1.4	2381	Season iii
G	Marlborough	Sauvignon Blanc/101-14/7 years	2.7 × 1.8	2058	Season iii
B	Hawke’s Bay	Riesling/Montana/SO_4_/15 years	2.5 × 2.25	1778	Only 1 season

^1^ Different ascending numbering systems are used for Chardonnay vs. Sauvignon Blanc to indicate that trials for both varieties were performed over consecutive seasons, but that season 1 in Chardonnay does not necessarily correspond to the same calendar year as season i in Sauvignon Blanc. ^2^ Cuttings taken from several vines of the same variety that have collectively demonstrated desired traits.

## Data Availability

The data presented in this study are contained within the article and Supplementary Material at www.mdpi.com/2223-7747/10/3/423/s1.

## Supplementary Materials

The following are available online at https://www.mdpi.com/2223-7747/10/3/423/s1, Table S1. Treatment spray schedule for field trials carried out on Chardonnay winegrapes in New Zealand over multiple successive seasons (indicated by incremental season numbers), where “-” means that sprays were not applied. Timing of each phenological stage of grape development in the Southern Hemsisphere is indicated in parentheses by the first 3 letters of the relevant calendar month. Table S2. Treatment spray schedule for field trials carried out on Sauvignon Blanc (SB) and Riesling winegrape varieties in New Zealand over multiple successive seasons (as shown by incremental roman numerals). The industry spray standard and integrated pest management (IPM) programmes are the same as described in Supplementary Table X.
