# Supplementary material for: Lipid-Based Natural Food Extracts for Effective Control of Botrytis Bunch Rot and Powdery Mildew on Field-Grown Winegrapes in New Zealand
Source: Plants (Basel). 2021 Feb 24;10(3):423. doi: 10.3390/plants10030423 (PMC7996311; doi:10.3390/plants10030423)
Supplement: Supplementary file 1 [file plants-10-00423-s001.pdf]

Table S1. Treatment spray schedule for field trials carried out on Chardonnay winegrapes in New Zealand over multiple successive seasons (indicated by incremental season numbers), where '–' means that sprays were not applied. Timing of each phenological stage of grape development in the Southern Hemsisphere is indicated in parentheses by the first 3 letters of the relevant calendar month.

Strategy	Treatment	Stage of Grapevine Phenology (and Approximate Month of the Year)								
		5–30 % Capfall (Nov/Dec)	80–95% Capfall (Dec)	Post-Bloom (Dec)	Berries Pea Size (Dec/Jan)	Pre-Bunch Closure (Jan)	Post-Bunch Closure Spray[1] (Jan/Feb)	Véraison (Feb)	4–6 Wk Pre-Vintage (Mar)	1–2 Weeks Pre-Vintage (Mar)
					**Season 1**					
Control	Unsprayed	–	–	–	–	–	–	–	–	–
PM Control	Kumulus	Kumulus® (30 g/10 L)+	Kumulus	Kumulus	Kumulus	Kumulus	Kumulus	Kumulus	–	–
Single Fungicide	Kocide	Kocide® (15 g/10 L)+	Kocide	Kocide	Kocide	Kocide	Kocide	Kocide	Kocide	Kocide
Industry Spray Standard[2]	Full fungicide[2]	Shirlan® (10 mL/10 L) & Kumulus	Switch® (8 g/10 L) & Kumulus	Dithane® (20 g/10 L) & Kumulus	Captan FLO (12.5 g/10 L) & Kumulus	Teldor® (7.5 mL/10 L) & Kumulus	Captan & Kumulus	Scala® (20 mL/10 L) & Kumulus	Captan FLO	Rovral® (300 mL/10 L)
NP	NP1	NP1 (7 g/L)+	NP1	NP1	NP1	NP1 (1 spray)	NP1	NP1	NP1	NP1
					**Season 2**					
Control	Nil botryticide	–	–	Topas® (1.25 mL/10 L)	–	Thiovit Jet® (3 kg/ha)	Thiovit Jet	–	–	–
Industry Spray Standard	Full fungicide	Euparen Multi® (2 kg/ha)	Switch (800 g/ha)	Topas	Captan (20 mL/ 10L)	Switch	Captan & Thiovit Jet	Captan	Captan	Captan
NP1 in IPM[3]	NP1	BOTRY-Zen® (4 kg/ha)	BOTRY-Zen	NP1 (7 g/L)	BOTRY-Zen	NP1	NP1	BCA-L1 (2 x 10^7 spores/mL)	BCA-L1	BCA-L1
NP Mix in IPM	NP1 early/NP2 mid	BOTRY-Zen	BOTRY-Zen	NP1	BOTRY-Zen	NP2 (15 g/L)	NP2	BCA-L1	BCA-L1	BCA-L1
NP2 in IPM	NP2	BOTRY-Zen	BOTRY-Zen	NP2 (15 g/L)	BOTRY-Zen	NP2	NP2	BCA-L1	BCA-L1	BCA-L1
					**Season 3**					
Control	Nil botryticide	–	–	Topas (1.25 mL/L)	–	–	–	–	–	–
Industry Spray Standard	Full fungicide	Euparen Multi (2 kg/ha)	Switch (800 g/ha)	Topas	Captan (20 mL/ 10L)	Switch	Captan (2 sprays)	Captan	Captan (2 sprays)	Captan

NP2 in IPM	NP2	BOTRY-Zen (4 kg/ha)	BOTRY-Zen	NP2 (5 g/L)	BOTRY-Zen	NP2	NP2 (2 sprays)	BCA-L1 (2 x 10^7 spores/mL)	BCA-L1 (2 sprays)	BCA-L1
					**Season 4**					
Control	Nil botryticide	–	–	Topas (1.25 mL/L)	–	Kumulus (3 kg/ha)	Kumulus or Topas (2 sprays)	–	–	–
Industry Spray Standard	Full fungicide	Euparen Multi (2 kg/ha)	Switch (800 g/ha)	Topas	Captan (20 mL/10 L)	Switch	Captan or Kumulus (2 sprays)	Captan	–	–
NP2 in IPM	BZ/NP2/BCA-L1	BOTRY-Zen (4 kg/ha)	BOTRY-Zen	NP2 (5 g/L)	BOTRY-Zen	NP2	NP2 (2 sprays)	BCA-L1 (2 x 10^7 spores /mL) (2 sprays)	BCA-L1	BCA-L1
Switch Instead of NP2	BZ/Switch/AZ	BOTRY-Zen	BOTRY-Zen	Toaps	BOTRY-Zen	Switch	Topas or Kumulus & ARMOUR-Zen® (5 L/ha) (2 sprays)	ARMOUR-Zen (2 sprays)	ARMOUR-Zen	ARMOUR-Zen
Commercially Available IPM Products	BZ/NP2/AZ	BOTRY-Zen (4 kg/ha)	BOTRY-Zen	NP2 (5 g/L)	BOTRY-Zen	NP2	NP2 (2 sprays)	ARMOUR-Zen (2 sprays)	ARMOUR-Zen	ARMOUR-Zen

[1] Unless stated otherwise, there is only 1 spray at each phenological stage. [2] The industry spray standard is the recommnded spray programme from the New Zealand grape industry for producing premium winegrapes under environmental conditions that favour Botrytis and PM outbreaks, and comprises application of a range of fungicides applied through the season – the registered names and rates of which are indicated at each phenological stage of grape development. Product rates do not change within a season, so the rates are not repeated when the same product is used more than once within a season. [3] After the initial season, the NPs were integrated into an Integrated pest management (IPM) programme, with BOTRY-Zen® (4 kg/ha formulation of the biocontrol agent, *Ulocladium oudemansii*) applied for part of the early-season (late-November to late-December), and BCA-L1, an experimental formulation of *Aureobasidium pullulans* (2 x 10^7 spores/mL) was applied during the late season (mid-February to mid-March). Avoiding use of the NPs post véraison in the IPM programmes mitigated the issue of lower °Brix at harvest in the NP treatments. NP2 has been commercialised is New Zealand as MIDI-Zen®.

Table S2. Treatment spray schedule for field trials carried out on Sauvignon blanc (SB) and Riesling winegrape varieties in New Zealand over multiple successive seasons (as shown by incremental roman numerals). The industry spray standard and integrated pest management (IPM) programmes are the same as described in Supplementary Table S1.

Strategy	Treatment	Stage of Grapevine Phenology (and Approximate Month of the Year)								
		5–30 % Capfall (Nov/Dec)	80–95% Capfall (Dec)	Post-Bloom (Dec)	Berries Pea Size (Dec/Jan)	Pre-Bunch Closure (Jan)	Post-Bunch Closure Spray (Jan/Feb)	Véraison (Feb)	4–6 Weeks Pre-Vintage (Mar)	2 Week Pre-Vintage (Mar)
**SB, Season i[1]**										
Control	Nil botryticide	–	–	Topas® (1.25 mL/L)	–	Kumulus® (3 kg/ha)	Kumulus (2 sprays)	–	–	–
Industry Spray Standard	Full fungicide	Euparen Multi® (2 kg/ha)	Switch® (800 g/ha)	Topas	Captan FLO (20 mL/10 L)	Switch & Kumulus	Captan & Kumulus (2 sprays)	Captan	Captan (2 sprays)	Captan
NP2 in IPM	NP2 (5 g/L)	BOTRY-Zen® (4 kg/ha)	BOTRY-Zen	Topas	BOTRY-Zen	NP2	NP2 (2 sprays)	BCA-L1 (2 x 10^7 spores/mL)	BCA-L1 (2 sprays)	BCA-L1
**SB, Season ii**										
Control	Nil botryticide	–	–	Topas (1.25 mL/L)	–	Kumulus (3 kg/ha)	Kumulus (2 sprays)	–	–	–
Industry Spray Standard	Full fungicide	Euparen Multi (2 kg/ha)	Switch (800 g/ha)	Topas	Captan (20 mL/10 L)	Switch & Kumulus	Captan & Kumulus (2 sprays)	Captan	Captan (2 sprays)	Rovral® (3 L/ha)
NP2 in IPM	BZ/NP2/BCA-L1	BOTRY-Zen (4 kg/ha)	BOTRY-Zen	Topas	BOTRY-Zen	NP2 (5 g/L)	NP2 (2 sprays)	BCA-L1 (2 x 10^7 spores /mL)	BCA-L1 (2 sprays)	BCA-L1
Switch Instead of NP2	BZ/Switch/AZ	BOTRY-Zen	BOTRY-Zen	Toaps	BOTRY-Zen	Switch	ARMOUR-Zen® (5 L/ha) (2 sprays)	ARMOUR-Zen	ARMOUR-Zen (2 sprays)	ARMOUR-Zen
Commercially Available IPM	BZ/NP2/AZ	BOTRY-Zen (4 kg/ha)	BOTRY-Zen	NP2 (5 g/L)	BOTRY-Zen	NP2	NP2 (2 sprays)	ARMOUR-Zen	ARMOUR-Zen (2 sprays)	ARMOUR-Zen
**SB, Season iii**										
Control, Standard Canopy Density (SCD)	Nil botryticides, SCD	–	–	–	–	Topas (1.25 mL/L)	–	–	–	–

Control, low Canopy Density (LCD)	Nil botryticides, LCD	–	–	–	–	Topas	–	–	–	–
NP2, SCD	NP2, SCD	Captan 80 WG (1.25 g/L)	Captan	–	NP2 (30 mL/L)[2]	NP2	–	NP2 (2 sprays)	Captan	Captan
NP2, LCD	NP2, LCD	Captan	Captan)	–	NP2	NP2	–	NP2 (2 sprays)	Captan	Captan
					**Riesling**					
Control	Nil botryticide	–	–	Topas (1.25 mL/10 L)	–	–	–	–	–	–
Industry Spray Standard	Full fungicide	Euparen Multi (2 kg/ha)	Switch (800 g/ha)	Topas	Captan (20 mL/10 L)	Switch & Thiovit Jet® (3 kg/ha)	Captan & Thiovit	Captan	Captan	Captan
NP1	NP1 (7 g/L)	BOTRY-Zen (4 kg/ha	BOTRY-Zen	Topas	NP1 (7 g/L)	NP1	NP1	NP1	NP1	NP1
NP2 Dose Response (DR)	NP2 (5 g/L)	BOTRY-Zen	BOTRY-Zen	Topas	NP2 (5 g/L)	NP2	NP2	NP2	NP2	NP2
NP2, DR	NP2 (10 g/L)	BOTRY-Zen	BOTRY-Zen	Topas	NP2 (10 g/L)	NP2	NP2	NP2	NP2	NP2
NP2, DR	NP2 (20 g/L)	BOTRY-Zen	BOTRY-Zen	Topas	NP2 (20 g/L)	NP2	NP2	NP2	NP2	NP2

[1] Different ascending numbering systems are used for Chardonnay (Supplementary Table X) vs Sauvignon blanc seasons to indicate that trials for both varieties were performed over consecutive seasons, but that season 1 in Chardonnay does not necessarily correspond to the same calendar year as season i in Sauvignon blanc. The trial on Riesling grapes was only performed in one season. [2] The formulation had to be changed because one of the co-ingredients, an anitooxidant, was taken off the market and had to replaced, hence a higher than normal concentration of the active ingredient (soybean oil) was used to ensure disease control efficacy.
